# Supplementary material for: Racial and ethnic disparities in longitudinal trajectories of cardiovascular risk factors in U.S. middle-aged and older adults
Source: PLoS One. 2025 Feb 7;20(2):e0318419. doi: 10.1371/journal.pone.0318419 (PMC11805438; doi:10.1371/journal.pone.0318419)
Supplement: S1 File — (PDF) [file pone.0318419.s001.pdf]

Supporting Information

Table of Contents

S1 Table. Baseline Risk Factors in U.S. Middle-Aged and Older Adults by Trajectory Group Membership (n=10,292) ____ Page 2

S2 Table. Baseline Characteristics of U.S. Middle-Aged and Older Adults by Trajectory Group Membership (n=10,292) ____Pages 3, 4

S1 Appendix. STROBE Statement—Checklist of items that should be included in reports of cohort studies____Pages 5, 6

S1 Table. Baseline Risk Factors in U.S. Middle-Aged and Older Adults by Trajectory Group Membership (n=10,292)

	**Group 1** (n=3,467)	**Group 2** (n=2,398)	**Group 3** (n=1,583)	**Group 4** (n=987)	**Group 5** (n=757)	**Group 6** (n=380)	**Group 7** (n=407)	**Group 8** (n=313)
**Risk Factors**	Mean (SD) or n (%)							
Systolic BP (mmHg)	116.6 (13.0)	138.9 (14.4)	122.1 (13.5)	126.7 (16.8)	147.1 (16.0)	131.6 (16.1)	131.5 (18.6)	170.6 (20.2)
Non-HDL cholesterol (mg/dL)	145.2 (36.1)	148.9 (35.2)	135.6 (34.9)	152.4 (40.5)	148.9 (39.0)	216.7 (38.4)	149.7 (40.6)	153.7 (42.4)
Total cholesterol (mg/dL)	203.8 (39.6)	203.5 (38.3)	186.8 (38.0)	206.6 (42.9)	200.2 (41.0)	272.2 (39.9)	198.6 (42.1)	208.8 (44.6)
HDL cholesterol (mg/dL)	58.6 (16.8)	54.8 (15.6)	51.2 (14.7)	54.2 (16.2)	51.4 (15.4)	55.5 (15.5)	48.9 (13.5)	55.1 (17.3)
Diabetes mellitus (y/n)	39 (1.1)	32 (1.3)	1260 (79.6)	8 (0.8)	630 (83.2)	44 (11.6)	305 (74.9)	60 (19.2)
Currently smoking (y/n)	79 (2.3)	51 (2.1)	57 (3.6)	953 (96.6)	16 (2.1)	26 (6.8)	383 (94.1)	122 (39.0)

Abbreviations: SD, standard deviation; BP, blood pressure; HDL, high-density lipoprotein.

S2 Table. Baseline Characteristics of U.S. Middle-Aged and Older Adults by Trajectory Group Membership (n=10,292)

	**Group 1** (n=3,467)	**Group 2** (n=2,398)	**Group 3** (n=1,583)	**Group 4** (n=987)	**Group 5** (n=757)	**Group 6** (n=380)	**Group 7** (n=407)	**Group 8** (n=313)
**Sociodemographic Factors**	Mean (SD) or n (%)							
Age (years)	62.0 (7.5)	62.8 (7.5)	61.9 (7.1)	60.5 (7.1)	62.7 (7.1)	62.2 (7.4)	59.7 (6.0)	62.5 (7.6)
Sex								
Male	823 (23.7)	1495 (62.3)	613 (38.7)	419 (42.5)	411 (54.3)	126 (33.2)	179 (44.0)	151 (48.2)
Female	2644 (76.3)	903 (37.7)	970 (61.3)	568 (57.6)	346 (45.7)	254 (66.8)	228 (56.0)	162 (51.8)
Race and Ethnicity								
Non-Hispanic White	2615 (75.4)	1761 (73.4)	842 (53.2)	662 (67.1)	357 (47.2)	259 (68.2)	193 (47.4)	151 (48.2)
Non-Hispanic Black	401 (11.6)	314 (13.1)	346 (21.9)	203 (20.6)	220 (29.1)	53 (14.0)	130 (31.9)	108 (34.5)
Hispanic	361 (10.4)	264 (11.0)	325 (20.5)	98 (9.9)	149 (19.7)	55 (14.5)	64 (15.7)	46 (14.7)
Non-Hispanic other	90 (2.6)	59 (2.5)	70 (4.4)	24 (2.4)	31 (4.1)	13 (3.4)	20 (4.9)	8 (2.6)
Nativity								
Foreign born	388 (11.2)	295 (12.3)	311 (19.7)	76 (7.7)	151 (20.0)	50 (13.2)	54 (13.3)	40 (12.8)
Native born	3079 (88.8)	2103 (87.7)	1272 (80.4)	911 (92.3)	606 (80.1)	330 (86.8)	353 (86.7)	273 (87.2)
Region								
South region	1312 (37.8)	926 (38.6)	698 (44.1)	419 (42.5)	337 (44.5)	158 (41.6)	175 (43.0)	138 (44.1)
Non-South region	2155 (62.2)	1472 (61.4)	885 (55.9)	567 (57.5)	420 (55.5)	222 (58.4)	232 (57.0)	175 (55.9)
Marital Status								
Never married	146 (4.2)	91 (3.8)	85 (5.4)	62 (6.3)	30 (4.0)	12 (3.2)	29 (7.1)	31 (9.9)
Married	2538 (73.2)	1829 (76.3)	1094 (69.1)	583 (59.1)	531 (70.1)	276 (72.6)	239 (58.7)	190 (60.7)
Divorced	446 (12.9)	294 (12.3)	246 (15.5)	226 (22.9)	113 (14.9)	50 (13.2)	93 (22.9)	50 (16.0)
Widowed	336 (9.7)	183 (7.6)	158 (10.0)	116 (11.8)	83 (11.0)	42 (11.0)	45 (11.1)	42 (13.4)
Educational Attainment								
Less than HS	364 (10.5)	326 (13.6)	326 (20.6)	228 (23.1)	188 (24.8)	63 (16.6)	120 (29.5)	98 (31.3)
HS degree	1116 (32.2)	849 (35.4)	518 (32.7)	383 (38.8)	275 (36.3)	150 (39.5)	156 (38.3)	110 (35.1)
Some college	909 (26.2)	572 (23.9)	414 (26.2)	266 (27.0)	174 (23.0)	90 (23.7)	101 (24.8)	78 (24.9)
College	1078 (31.1)	651 (27.1)	325 (20.5)	109 (11.1)	120 (15.9)	77 (20.3)	30 (7.4)	27 (8.6)
HH income (per $10k)	8.7 (12.1)	8.6 (12.3)	6.2 (9.1)	4.9 (5.4)	5.4 (5.6)	7.4 (9.6)	4.0 (4.1)	4.8 (6.1)
**(Cont.)**								

Abbreviations: SD, standard deviation; HS, high school; HH: household

S2 Table (cont.). Baseline Characteristics of U.S. Middle-Aged and Older Adults by Trajectory Group Membership (n=10,292)

	Group 1 (n=3,467)	Group 2 (n=2,398)	Group 3 (n=1,583)	Group 4 (n=987)	Group 5 (n=757)	Group 6 (n=380)	Group 7 (n=407)	Group 8 (n=313)
**Health-Related Factors**	Mean (SD) or N (%)							
Physical inactivity								
Sedentary	1690 (48.8)	1057 (44.1)	953 (60.2)	593 (60.1)	456 (60.2)	207 (54.5)	275 (67.6)	174 (55.6)
Non-sedentary	1774 (51.2)	1341 (55.9)	630 (39.8)	394 (39.9)	299 (39.5)	172 (45.3)	132 (32.4)	137 (43.8)
Body Mass Index (BMI)								
Underweight	76 (2.2)	30 (1.3)	29 (1.8)	27 (2.7)	16 (2.1)	3 (0.8)	13 (3.2)	8 (2.6)
Normal weight	1102 (31.8)	535 (22.3)	179 (11.3)	377 (38.2)	60 (7.9)	60 (15.8)	66 (16.2)	74 (23.6)
Overweight	1286 (37.1)	1038 (43.3)	468 (29.6)	368 (37.3)	241 (31.8)	154 (40.5)	150 (36.9)	112 (35.8)
Obese	1003 (28.9)	795 (33.1)	907 (57.3)	215 (21.8)	440 (58.1)	163 (42.9)	178 (43.7)	119 (38.0)
Alcohol Consumption								
Never	2075 (59.8)	1268 (52.9)	1193 (75.4)	526 (53.3)	548 (72.4)	252 (66.3)	270 (66.3)	178 (56.9)
Moderate	1181 (34.1)	796 (33.2)	283 (17.9)	227 (23.0)	138 (18.2)	93 (24.5)	79 (19.4)	78 (24.9)
Heavy	211 (6.1)	331 (13.8)	106 (6.7)	231 (23.4)	70 (9.3)	34 (9.0)	55 (13.5)	57 (18.2)
Depressive symptoms	1.2 (1.8)	1.1 (1.7)	1.6 (2.1)	1.9 (2.3)	1.6 (2.1)	1.6 (2.1)	2.6 (2.4)	1.7 (2.1)
CVD Medications								
Anti-hypertensives	1320 (38.1)	1062 (44.3)	1030 (65.1)	356 (36.1)	536 (70.8)	165 (43.4)	243 (59.7)	172 (55.0)
Statins	1092 (31.5)	792 (33.0)	933 (58.9)	267 (27.1)	421 (55.6)	141 (37.1)	211 (51.8)	89 (28.4)
Diagnosed Conditions								
Psychiatric disorder	521 (15.0)	269 (11.2)	287 (18.1)	224 (22.7)	117 (15.5)	76 (20.0)	116 (28.5)	35 (11.2)
Heart disease	509 (14.7)	331 (13.8)	342 (21.6)	137 (13.9)	168 (22.2)	49 (12.9)	104 (25.6)	41 (13.1)
Stroke or TIA	132 (3.8)	86 (3.6)	73 (4.6)	47 (4.8)	55 (7.3)	15 (4.0)	43 (10.6)	29 (9.3)
COPD	201 (5.8)	126 (5.3)	125 (7.9)	149 (15.1)	42 (5.6)	26 (6.8)	73 (17.9)	19 (6.1)
Cancer	377 (10.9)	240 (10.0)	179 (11.3)	73 (7.4)	61 (8.1)	42 (11.1)	33 (8.1)	24 (7.7)
Missing	18	14	7	6	7	5	7	3

Abbreviations: SD, standard deviation; TIA, transient ischemic attack; COPD, chronic obstructive pulmonary disease.

S1 Appendix. STROBE Statement—Checklist of items that should be included in reports of cohort studies

	Item No	Recommendation	Page No
**Title and abstract**	1	(*a*) Indicate the study's design with a commonly used term in the title or the abstract<br>(*b*) Provide in the abstract an informative and balanced summary of what was done and what was found	Page 2<br>Page 2
**Introduction**			
Background/rationale	2	Explain the scientific background and rationale for the investigation being reported	Page 3
Objectives	3	State specific objectives, including any prespecified hypotheses	Page 4
**Methods**			
Study design	4	Present key elements of study design early in the paper	Page 4
Setting	5	Describe the setting, locations, and relevant dates, including periods of recruitment, exposure, follow-up, and data collection	Pages 4, 5
Participants	6	(*a*) Give the eligibility criteria, and the sources and methods of selection of participants. Describe methods of follow-up<br>(*b*) For matched studies, give matching criteria and number of exposed and unexposed	Page 4<br>N/A
Variables	7	Clearly define all outcomes, exposures, predictors, potential confounders, and effect modifiers. Give diagnostic criteria, if applicable	Pages 5, 6
Data sources/ measurement	8*	For each variable of interest, give sources of data and details of methods of assessment (measurement). Describe comparability of assessment methods if there is more than one group	Pages 5, 6
Bias	9	Describe any efforts to address potential sources of bias	N/A
Study size	10	Explain how the study size was arrived at	Page 4
Quantitative variables	11	Explain how quantitative variables were handled in the analyses. If applicable, describe which groupings were chosen and why	Pages 5, 6
Statistical methods	12	(*a*) Describe all statistical methods, including those used to control for confounding<br>(*b*) Describe any methods used to examine subgroups and interactions<br>(*c*) Explain how missing data were addressed<br>(*d*) If applicable, explain how loss to follow-up was addressed<br>(*e*) Describe any sensitivity analyses	Pages 6-8
**Results**			
Participants	13*	(a) Report numbers of individuals at each stage of study—eg numbers potentially eligible, examined for eligibility, confirmed eligible, included in the study, completing follow-up, and analysed<br>(b) Give reasons for non-participation at each stage<br>(c) Consider use of a flow diagram	N/A

Descriptive data	14*	(a) Give characteristics of study participants (eg demographic, clinical, social) and information on exposures and potential confounders	Page 8, Table 1
		(b) Indicate number of participants with missing data for each variable of interest	eTable 2
		(c) Summarise follow-up time (eg, average and total amount)	Page 4
Outcome data	15*	Report numbers of outcome events or summary measures over time	eTable 1

Main results	16	(*a*) Give unadjusted estimates and, if applicable, confounder-adjusted estimates and their precision (eg, 95% confidence interval). Make clear which confounders were adjusted for and why they were included	Pages 8-10 Tables 2-5
		(*b*) Report category boundaries when continuous variables were categorized	
		(*c*) If relevant, consider translating estimates of relative risk into absolute risk for a meaningful time period	
Other analyses	17	Report other analyses done—eg analyses of subgroups and interactions, and sensitivity analyses	N/A
**Discussion**			
Key results	18	Summarise key results with reference to study objectives	Page 10
Limitations	19	Discuss limitations of the study, taking into account sources of potential bias or imprecision. Discuss both direction and magnitude of any potential bias	Page 12, 13
Interpretation	20	Give a cautious overall interpretation of results considering objectives, limitations, multiplicity of analyses, results from similar studies, and other relevant evidence	Pages 10-12
Generalisability	21	Discuss the generalisability (external validity) of the study results	Page 12, 13
**Other information**			
Funding	22	Give the source of funding and the role of the funders for the present study and, if applicable, for the original study on which the present article is based	Page 14

*Give information separately for exposed and unexposed groups.

Note: An Explanation and Elaboration article discusses each checklist item and gives methodological background and published examples of transparent reporting. The STROBE checklist is best used in conjunction with this article (freely available on the Web sites of PLoS Medicine at http://www.plosmedicine.org/, Annals of Internal Medicine at http://www.annals.org/, and Epidemiology at http://www.epidem.com/). Information on the STROBE Initiative is available at http://www.strobe-statement.org.
