# Supplementary material for: Racial and ethnic disparities in longitudinal trajectories of cardiovascular risk factors in U.S. middle-aged and older adults
Source: PLoS One. 2025 Feb 7;20(2):e0318419. doi: 10.1371/journal.pone.0318419 (PMC11805438; doi:10.1371/journal.pone.0318419)
Supplement: Attachment — Submitted filename: Response to Reviewers.docx [file pone.0318419.s002.docx]

**Reviews and Comments**

We would like to thank the Editor and Reviewers for their thoughtful comments and for giving us the opportunity to address these concerns in a revised submission. As you will see, we took this guidance very seriously and address each of the concerns in this memo and in the revised manuscript. In doing so, we believe the study is much improved. We respond separately to each comment below.

**Reviewer #1:**

**This is a carefully thought-out analyses and the authors were successfully able to draw new data that highlights covariates that mediate disparities of racial and ethnic minorities (in middle-aged and older adults) in relation to their risks for different CVD risk factors, placing them in different trajectory group membership. Most of key data was highlighted and the methods were clear. The study is of significance to the broader scientific community. I only have small suggestions here.**

**1. Tables 4 and 5 were mostly utilized to demonstrate the sociodemographic factors that would place Hispanic adults or non-Hispanic black adults in different group memberships. It seems health related factors were not really mentioned and discussed much in text, whether in results / discussion. It might be more suitable for discussion. What is the relationship between the health-related factors and the sociodemographic factors (if any)? Or simply acknowledge that looking at Table 4 and Table 5, health-related factors can account for some CVD risks, 36% for Hispanic adults, and 25% for non-Hispanic black adult.**

RESPONSE: Thank you for this helpful suggestion. We agree with your suggestion to discuss the health-related factors in Tables 4 and 5 and have included text in the discussion section (page 22) to note that, “among health-related factors, obesity and depressive symptoms played a significant role in their association with suboptimal trajectories of CVD risk factors in both non-Hispanic Black adults (up to 24%) and Hispanic adults (up to 36%).”

**2. Along the lines of the previous paragraph, can the study comment on baseline health characteristics of non-Hispanic black or Hispanic minorities compared to non-Hispanic white adults? These do not have to necessarily be mentioned with regards to CVD risks, but it would be important as health-related factors are also important covariates to consider in such analyses, whether in context of this study or previous studies.**

RESPONSE: Thank you for this suggestion. Table 1 provides the baseline characteristics of the study population by racial and ethnic groups. In terms of health-related factors, non-Hispanic Black participants and Hispanic participants were more likely to be obese at baseline and reported greater sedentary behavior, more depressive symptoms, and use of statins and antihypertensive medications, compared to other racial and ethnic groups (non-Hispanic White and non-Hispanic Other).

**Reviewer #2**:

**1. Please expand the introduction section to shed light on ethnicity and racial background as a determinant for population health, why certain ethnic/ racial groups are considered a health disparity.**

RESPONSE: Thank you for raising this point. On page 4 (introduction section, first paragraph), we describe that there is a higher prevalence of cardiovascular disease (CVD) in racial and ethnic minority groups due to the underlying disparities in the prevalence and management of CVD risk factors in these populations. We further expand on this point by highlighting the socioeconomic disadvantages that predispose certain racial and ethnic groups to poor health outcomes (page 4).

**2. Define the rationale of the study**

RESPONSE: Thank you for allowing us to clarify. In the introduction section, we specify the rationale and main objectives of the study (see second and third paragraph). Briefly, previous studies that focus on racial and ethnic disparities in CVD risk typically focus on individual risk factors and do not consider the interplay among multiple risk factors, nor do they account for the progression of these risk factors with increasing age. To address this knowledge gap, our study describes how CVD risk factors co-occur and vary with age and across individuals and highlights the need for targeting preventive care and tailoring risk-reduction strategies in middle-aged and older adults.

**3. Identify the impact of the study at the population and clinical practice levels.**

RESPONSE: Thank you for this important comment. Throughout the discussion section, we identify the impact of our findings at both the population and clinical practice levels. On page 20, we highlight the complex interplay between multiple CVD risk factors and the corresponding need for “aggressive lifestyle and/or pharmacological interventions to mitigate these compounding risk factors” and tailored clinical decision-making for select populations through “more intensive prevention and management strategies and multidisciplinary care coordination.” Furthermore, on page 21, we discuss how our findings may be helpful for addressing the disparities faced by certain racial and ethnic groups by “identifying the timing and combinations of these risk factors as vital for informing preventive care efforts and risk-reduction strategies—*e.g.*, when to prioritize lifestyle counseling vs. pharmacological interventions—in racial and ethnic minority populations.” Additionally, we reiterate the need for “socially and culturally-relevant approaches as critical for implementing self-management strategies to reduce CVD risks and improving patient outcomes” in certain racial and ethnic groups. Finally, on page 22, we underscore the importance of social determinants of health in affecting health outcomes and suggest that “healthcare providers should remain vigilant in monitoring racial and ethnic minority groups—especially those who identify as Hispanic, foreign-born (or non-native origin), and those with lower educational attainment and income—for initiating timely and tailored interventions based on their risk profile.”

**4. Because the SD in the age is pretty wide which indicates presence of outliers, please use the median instead.**

RESPONSE: Thank you for this important suggestion. We have included the median (interquartile range) for age in the abstract, results section, and in Table 1.

**5. Define "South" and explain why South had been a classification category.**

RESPONSE: Thank you for allowing us to clarify. The South is a geographic region defined by the U.S. Census Bureau and is the largest region compared to the other three census regions (Northeast, Midwest, and West). More notably, South was used as a classification category because of known geographic differences associated with greater CVD risks in the southern United States. As suggested, we have included a sentence in the methods section (Page 7) to clarify why South was used as a classification category.

**6. Expand the discussion section to further correlate between the findings of this study and previous studies that had assessed similar populations.**

RESPONSE: Thank you for this helpful suggestion. We cite several research studies whose findings correlate with the findings of our study. For example, in the discussion section (page 20), we include studies that describe the complex interactions between multiple CVD risk factors: “These findings underscore the importance of considering the complex interplay among multiple risk factors that are observed among U.S. middle-aged and older adults (5-7)”. Further in the discussion (page 21), we cite studies that describe the racial and ethnic disparities prevalent in cardiovascular disease outcomes in the US: “Our study also identified significant racial and ethnic disparities in the age-related trajectories of CVD risk factors (2)” and how these disparities may be best targeted with “integrating socially and culturally-relevant approaches is critical for implementing self-management strategies to reduce CVD risks and improving patient outcomes (28-30).” We also correlate our findings on the social determinants of health with other studies. Specifically, “our study provides new evidence to support this body of work and demonstrates that socioeconomic factors played a significant role in the associations between race and ethnicity and trajectory group membership (2)” and how “country of origin was another key factor in the likelihood that Hispanic adults (born outside the U.S.) exhibited higher-risk CVD profiles relative to non-Hispanic White adults (31).” Finally, please note that our study is the first prospective investigation of racial and ethnic disparities in age-related trajectories of *multiple CVD risk factors* among U.S. middle-aged and older adults. Per your suggestion, we also acknowledge this in the limitations section (page 22): “… we encourage studies to further assess the patterns of additional (or different) trajectories of CVD risk factors that may be observed in other datasets/populations.” Lastly, in the conclusions section we state that, “additional studies, particularly among racial and ethnic minority populations are needed to confirm our findings.”

**7. Add a section for future recommendations.**

RESPONSE: Thank you for this suggestion. As noted in our response to Comment #3, we highlight the clinical and population-level implications based on our findings. In addition, the concluding section further summarizes these recommendations. Lastly, we included text in the discussion (page 22) highlighting the need to replicate these findings in racial and ethnic minority populations.

**Reviewer #3:**

**Thank you for the opportunity to review this manuscript in which the authors elucidate racial and ethnic disparities in longitudinal trajectories of cardiovascular risk factors in middle-aged and older adults in the United States. This is an exceptional manuscript. The study is well conceived, well executed, and well reported. I just have some minor comments for consideration for this or future work.**

**1. How might geographic location of participant residential address play a part, for example poor vs wealthy suburbs, and rurality? These play significant roles in access to health care and therefore health outcomes. I believe that the United States has an area deprivation index – I wonder if this might help address this aspect, or if there is a geographic classification for health?**

RESPONSE: Thank you for these valuable comments. We agree that geographic location may play a significant role in healthcare disparities. Indeed, neighborhood-level indicators such as the area deprivation index (ADI) would be a useful variable to address the role of geography in health outcomes. However, a participant’s 9-digit zip code (or Federal Information Processing Standard code [FIPS code]) is needed to link to their neighborhood’s ADI, and the available dataset for this study lacks such granular information. Therefore, we acknowledge the lack of information on geographic location and its association with health outcomes in the limitations section.

**2. Similarly, consideration of participant visa/residency status may shed further light on access to health care and therefore health outcome.**

RESPONSE: Thank you for this comment. Please note that the Health and Retirement Study lacks measures on the participants’ visa/residency status.

**3. For several variables, data is based on participant self-report of physician diagnosis. This presumes access to health providers, health literacy, and acceptability of diagnosis. Some discussion of the direction and magnitude of any influence on the results this might have is warranted in the Strengths & Limitations section.**

RESPONSE: Thank you for this important point. As suggested, we have included text in the limitations section about self-reported medical history and its possible impact on our findings.

**4. Very minor, in the Abstract: missing ‘[to] exhibit high rates of smoking’, and Nativity would benefit from explanation – it is explained in the Introduction, but it was a novel term to come across in the abstract.**

RESPONSE: Thank you for noticing the missing word, we have revised the sentence accordingly in the abstract. As suggested, we have also changed the term nativity to country of origin in the abstract to help simplify the interpretation of our findings.
